# Non-invasive exposure biomarkers of tobacco smoke exposure in smokers of classic cigarettes and users of e-cigarettes and heated tobacco products

**DOI:** 10.3389/fmolb.2025.1675523

**Published:** 2025-12-04

**Authors:** Kamila Jończyk, Aleksandra Kicman, Anna Michalska-Falkowska, Justyna Śniadach, Napoleon Waszkiewicz

**Affiliations:** 1 Department of Psychiatry, The Faculty of Medicine, Medical University of Bialystok, Bialystok, Poland; 2 MUB Biobank, Medical University of Bialystok, Bialystok, Poland

**Keywords:** classic cigarettes, e-cigarettes, heated tobacco products, biomarkers of exposure, cytokines, chemokines, oxidative stress parameters, urid biomarkers and saliva biomarkers

## Abstract

Exposure biomarkers are measurable biological indicators that indicate whether or not the body has been exposed to a particular chemical and the extent of that exposure. Exposure biomarkers are widely used in smokers. Today, there is a growing number of users of various forms of tobacco, especially in the form of e-cigarettes and heated tobaccos. The method of tobacco delivery has an impact on toxicity and biomarker concentrations. Therefore, it is expedient to introduce new biomarkers of tobacco smoke exposure, in addition to existing markers. The ideal biomarker is characterized by non-invasive intake - therefore saliva and urine should be considered as ideal material for determination of biomarkers of exposure. This paper summarizes the existing knowledge of classical and modern biomarkers of tobacco smoke exposure determined from urine and saliva and a brief overview on exposure biomarkers. In addition, the paper provides a description of future developments of exposure biomarkers in different groups of cigarette users.

## 1 Introduction

Smoking is one of the most important causes of disease ascension, and importantly these diseases are preventable. The number of deaths related to smoking-related diseases exceeds 8 million per year for smokers and 1.3 million for passive smokers (Kaufman et al., 2020; Rigotti et al., 2022). Cardiovascular diseases, lung diseases and cancer are cited as the most common smoking-related diseases (Gallucci et al., 2020, Elicker et al., 2019; Pichandi et al., 2011). Smokers also have a higher risk of respiratory tract infections, both bacterial and viral (Jiang et al., 2020; Ronsard et al., 2014; Ronsard et al., 2015). Today, the number of classic cigarette smokers has declined in favor of users of alternative tobacco methods such as e-cigarettes and heated tobacco products (HTPs) (Śniadach et al., 2025, Bitzer et al., 2020; Laverty et al., 2021). The highest number of smokers is observed between the ages of 20–39. The frequency of smoking is influenced by a number of factors such as attitude and type of work, stress in the profession and personal life, level of earnings, having offspring and education (Śniadach et al., 2025; Perkins et al., 2002). In general, females smoke more often which is due to the response to negative emotions, while males smoke due to pharmacological stimuli. It should be noted, however, that females metabolize nicotine more rapidly, making them more likely to use cigarettes compared to men (Śniadach et al., 2025; Perkins et al., 2002; Perkins et al., 2002). More than 70% of smokers are willing to quit, however, many of them do not maintain abstinence from smoking within a month of quitting (Suzuki et al., 2016). According to some studies, more than 98% of smokers return to smoking within a year of quitting (Suzuki et al., 2016; Kaufman et al., 2020). Limiting smoking (to 1–4 cigarettes per day) can reduce the risk of respiratory diseases and selected cancers, however, cardiovascular risk remains unchanged (Habibagahi et al., 2020).

Biomarkers are substances that allow assessment from exposure to a dose to the biological effects of taking that dose. An effective biomarker of exposure can be used when methods to directly measure external exposure are lacking (Shilnikova et al., 2022, Hiler et al., 2023; Saulyte et al., 2014). Exposure biomarkers can be detected in urine, saliva, hair or blood (Brucker et al., 2020; Martinez-Morata et al., 2023). In particular, biomarkers extracted by non-invasive methods, i.e., without disturbing tissue continuity, are highly useful - such material can be hair, tears, sweat, urine and saliva. In addition to non-invasive acquisition, they are also characterized by a relatively easy method of acquisition. Unlike urine and saliva, hair, sweat and tear collection are a minimally invasive method.

The physical properties of tobacco delivery systems can affect the toxicity and strength of tobacco addiction. It therefore seems advisable to consider new biomarkers of exposure other than nicotine or cotinine alone to assess exposure to tobacco products and tobacco smoke. This paper summarizes previous data on changes in the concentrations of new and classical biomarkers in non-invasively collected test material - saliva and urine in smokers.

## 2 Materials and methods

### 2.1 Search strategy

Independent authors screened titles and abstracts for relevance. Articles were searched in the following databases: PubMed, Scopus, Web of Science, and Google Scholar. The search included articles published in English. The literature taken was from 1950-2025. The following keywords were considered in the search strategy: “4-(methylnitrosamino)-1-(3-pyridyl)-1-butanol,” “Classic cigarettes,” “Cotinine,” “E-cigarettes,” “NNAL,” “Non-smokers,” “Smokers,” “Thiocyanate,” “(IL-1β),” “1-Hydroxypyrene,” “4-HNE,” “Catalase,” “CXCL8,” “GPx,” “HTP,” “IL-10,” “IL-13,” “IL-17,” “IL-23,” “IL-4,” “IL-6,” “IL-8,” “MCP-1,” “MDA,” “ROS,” “SOD,” “TGF-β,” “TNF-α,” “Uric acid”, “urine biomarkers,” “saliva biomarkers.” The selection of compound data for the paper was chosen based on the analysis of source data.

### 2.2 Inclusion criteria

We included clinical studies, reviews, meta-analyses, and case reports focusing on biomarkers of electronic cigarettes, classical cigarettes and heated tobacco products. Only studies that assessed biomarkers in saliva, or urine of human participants were considered eligible. To ensure the currency of our analysis, we prioritized the most recent publications. Additional inclusion criteria were: publication within the last 15 years, a minimum sample size of 50 participants (due to the reliability of scientific data), and the availability of detailed methodological information, including control conditions and statistical analyses.

### 2.3 Article selection process

**FIGURE 1:**
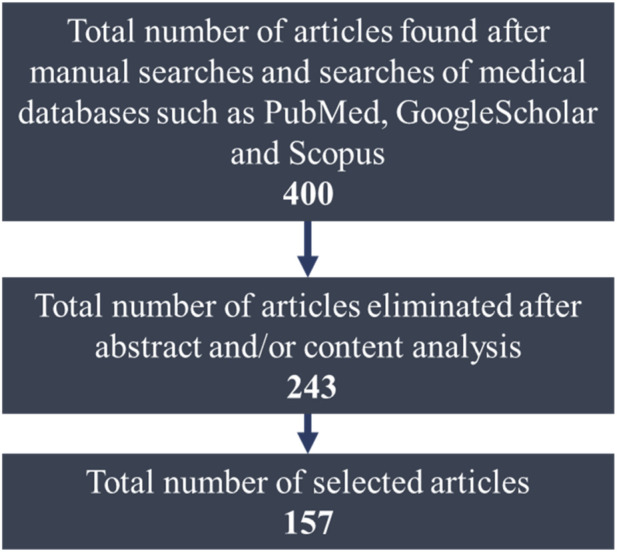
Flowchart of articles selection process.

The initial search identified a total of 400 articles. Following the application of the predefined inclusion and exclusion criteria, 141 articles were selected for further analysis. These studies were assessed with particular attention to sample size and the presence of control conditions to evaluate the reliability and validity of the findings. Detailed methodological characteristics, including sample sizes and control conditions, are reported within the respective sections of the selected studies. This is shown in Figure 1.

## 3 Exposure biomarkers–overview

Exposure biomarkers are measurable biological indicators that indicate whether or not an organism has been exposed to a particular chemical and to what extent that exposure has occurred (Shilnikova, et al., 2022; Hiler et al., 2023). As mentioned in the introduction, they can be determined in different types of biological material (Brucker et al., 2020; Martinez-Morata et al., 2023; Elicker et al., 2019). The presence of elevated or decreased concentrations of a particular biomarker in body fluids or tissues indicates that the body has been exposed to a particular chemical such as tobacco smoke, heavy metals, pesticides, drugs, narcotics or environmental pollutants. There are two types of biomarkers: biomarkers of exposure (which determine whether an organism has been exposed to a given factor) and biomarkers of potential harm (which determine the short-term health effects on the organism). Biomarkers can measure concentrations of chemicals or their metabolites, internal dose or exposure levels (Barbosa et al., 2005; Shilnikova, et al., 2022; Shilnikova, et al., 2025; Lee et al., 2025). Key examples of biomarker-substance relationships are shown in Table 1. Applications of exposure biomarkers include: 1) assessing environmental and occupational exposures to specific chemicals, 2) evaluating the effectiveness of health interventions, 3) performing epidemiological studies, 4) regulation for public health (Barbosa et al., 2005; Shilnikova, et al., 2022).

**TABLE 1:** Examples of biomarkers of exposure to various chemical compounds. Based on: Barbosa et al. (2005), Shilnikova, et al. (2022), Shilnikova, et al. (2025), Hiler et al. (2023), Biomarkers and Risk Assessment (2025).

Substance	Exposure biomarker
Benzene	S-phenylmercapturic acid
Lead	Lead concentration
Styrene	Styrene oxide
Parabens	2-ethoxyacetic acid and 2-ethoxyethanol
Mercury	Total mercury concentration
Carbon monoxide	Carboxyhemoglobin
Aromatic compounds (PAHs)	1-hydroxypyrene

Exposure biomarkers have advantages and disadvantages. Most of them are the high sensitivity of the compounds; relatively low concentrations of a given compound are sufficient for detection. Exposure biomarkers are also objective to a degree better than questionnaires collected from people exposed to harmful substances, and the ability to analyze current or cumulative or chronic exposure (Protano et al., 2024; Mayeux, 2004, Rodríguez-Rabassa et al., 2018). It is unfortunate that some biomarkers have short half-lives and require complex analytical methods for determination. Additionally, some have low specificity (Hays et al., 2007; Sexton, 1991).

## 4 Classical biomarkers of exposure in smokers

### 4.1 Nicotine

Nicotine is an organic chemical compound, an alkaloid that is found in tobacco, particularly in the leaves and roots of fine tobacco. Due to its nicotine content, tobacco is one of the most addictive biological substances (Martin and Sayette, 2018). Paradoxically, the amount of data on nicotine concentrations in urine and saliva is limited. Smokers of regular cigarettes had higher concentrations of nicotine in urine compared to non-smokers (Behera et al., 2003; Feyerabend et al., 1982, Benowitz et al., 2020; Feng et al., 2022; Pamungkasningsih et al., 2021; Taufik et al., 2021). Also, higher concentrations of nicotine are found in the unstimulated saliva of cigarette smokers (Behera et al., 2003; Feyerabend et al., 1982, Fallatah et al., 2018; Robson et al., 2010).

More than twice as much nicotine concentrations are found in the urine of e-cigarette smokers compared to non-smokers (people who do not use any form of tobacco) (Lorkiewicz et al., 2019). A single urine study of HTP users also found higher nicotine concentrations compared to non-users (people who do not use any form of tobacco) (Liu et al., 2024). Changes in nicotine and other biomarkers concentrations are described in Table 2.

**TABLE 2:** A summary of the effects of cigarette smoking, e-cigarette use and HTP use on biomarker levels in saliva and urine.

Biomarker	Cigarette smoking	E-cigarettes	HTP	Literature
*Nicotine*	Urine: Increase Saliva: Increase	Urine: Increase Saliva: Increase	Urine: Increase Saliva: Increase	Behera et al., 2003; Feyerabend et al., 1982, Benowitz et al., 2020; Feng et al., 2022; Pamungkasningsih et al., 2021; Fallatah et al., 2018; Robson et al., 2010
*Cotinine*	Urine: Increase Saliva: Increase	Urine: No data Saliva: No data	Urine: No data Saliva: No data	Sharma et al., 2019; Van Overmeire et al., 2016; Jung et al., 2012; Zielińska-Danch et al., 2007; Göney et al., 2016; Jain, 2015; Paci et al., 2018; Yang et al., 2001; Hovanec et al., 2019; Fernandes et al., 2020; Zettergren et al., 2023; Etter and Bullen, 2011; Hasan et al., 2024; Mokeem et al., 2018
*1-Hydroxypyrene*	Urine: Increase or no differences Saliva: No data	Saliva: Increase Saliva: No data	Urine: No data Saliva: No data	Carmella et al., 2003; Hou et al., 2012; Benowitz et al., 2018; Xia et al., 2011; Kavvadias et al., 2009; Dai et al., 2022; Edmiston et al., 2022
*4-(methylnitrosamino)-1-(3-pyridyl)-1-butanol (NNAL)*	Urine: Increase Saliva: No data	Urine: Increase Saliva: No data	Urine: Increase Saliva: No data	Hou et al., 2012; Kavvadias et al., 2009
*Interleukin 1β*	Urine: Similar concentration Saliva: Increase	Urine: Similar concentration Saliva: Increase, decrease or similar	Urine: No data Saliva: No data	Farell et al., 2023; Kamal and Shams, 2022; Suzuki et al., 2016; Zięba et al., 2024; Alhumaidan et al., 2022; Galanti, 1997; Rodríguez-Rabassa et al., 2018; Flieger et al., 2019; Prakruthi et al., 2018; Benny and D’Cruz, 2020; Nowicki et al., 1984; Satoh et al., 2015
*Interleukin 4*	Saliva: Increase Urine: No data	Urine: No data Saliva: No data	Urine: No data Saliva: No data	Farrell et al., 2023
*Interleukin 6*	Urine: Similar concentration Saliva: Similar concentration	Urine: Increase Saliva: Increase	Urine: No data Saliva: No data	Farrell et al., 2023; Singh et al., 2019; Verma et al., 2021; Rodríguez-Rabassa et al., 2018; Mokeem et al., 2018
*Interleukin 8*	Urine: Similar concentrations Saliva: Increase, decrease or similar	Saliva: Increase or decrease Urine: No data	Saliva: Decrease Urine: No data	Farrell et al., 2023; Zięba et al., 2024; Frasheri et al., 2022; Karaaslan et al., 2020; Sahibzada et al., 2023; Amirthalingam et al., 2023; Suzuki et al., 2016
*Interleukin 10*	Urine: Similar concentration Saliva: Decrease	Urine: Decrease or similar Saliva: Increase	Saliva: Increase Urine: No data	Satoh et al., 2015; Suzuki et al., 2016; Mokeem et al., 2018; Zięba et al., 2024; Khan et al., 2020
*Interleukin 13*	Urine: Increase Saliva: Increase or similar	Urine: Similar concentration Saliva: Increase	Urine: Similar concentration Saliva: Increase or similar	Farrell et al., 2023; Tanni et al., 2010; Zięba et al., 2024; Rodríguez-Rabassa et al., 2018; Kamal and Shams, 2022; Suzuki et al., 2016; Tanni et al., 2010; Barbieri et al., 2011
*Interleukin 17*	Saliva: Increase or similar Urine: No data	Urine: No data Saliva: No data	Urine: No data Saliva: No data	Rodríguez-Rabassa et al, 2018
*Interleukin 23*	Urine: Increase Saliva: No data	Urine: No data Saliva: No data	Urine: No data Saliva: No data	Barjaktarevic et al., 2016
*Tumor Necrosis Factor Alpha*	Saliva: Similar concentrations Urine: No data	Saliva: Increase Urine: No data	Saliva: Increasev Urine: No data	Suzuki et al., 2016, Frasheri et al., 2022; Belkin et al., 2023
*Monocyte Chemoattractant Protein 1*	Saliva: Decrease Urine: No data	Saliva: Decrease Urine: No data	Saliva: Decrease Urine: No data	Suzuki et al., 2016
*Transforming Growth Factor β*	Saliva: Increase	Saliva: Increase	Saliva: Decrease Urine: No data	Nowicki et al., 1984; Kamal and Shams, 2022
Glutathione peroxidase	Saliva: Increase, decrease or similar Urine: No data	Urine: No data Saliva: No data	Urine: No data Saliva: No data	Arbabi-Kalati et al., 2017; Saggu et al., 2012; Jenifer et al., 2015; Syed et al., 2021; Balasubramaniam and Arumugham, 2022; Zappacosta et al., 2002
Superoxide dismutase	Saliva: Decrease Urine: No data	Saliva: Decrease Urine: No data	Saliva: Increase or similar Urine: No data	Saggu et al., 2012; Syed et al., 2021; Suvarna et al., 2023
Catalase	Saliva: Decrease Urine: No data	Saliva: Decrease Urine: No data	Saliva: Decrease Urine: No data	Singh et al., 2019; Jenifer et al., 2015, Balasubramaniam and Arumugham, 2022; Ahmadi-Motamayel et al., 2018
*Malondialdehyde*	Saliva: Increase Urine: No data	Saliva: Increase Urine: No data	Saliva: Increase Urine: No data	Zięba et al., 2024, Demirtaş et al., 2014; Mohammed et al., 2014
*4-Hydroxynonenal*	Urine: Increase Saliva: No data	Urine: Increase Saliva: Increase	Saliva: Decrease Urine: No data	Zięba et al., 2024; Shoeb et al., 2014; Li et al., 2022; Eskelinen et al., 2022; Zięba et al., 2024; Žarković et al., 2024; Majid, 2024
Uric acid	Urine: Increase Saliva: Increase or similar	Saliva: Decrease Urine: No data	Saliva: Decrease Urine: No data	Miguel et al., 2022; Zarabadipour et al., 2022

### 4.2 Cotinine

Cotinine is the main metabolite of nicotine that is formed in the kidneys, lungs and liver. This compound is used to assess nicotine exposure because it is produced only during the metabolism of the compound. Additionally, it is quite stable and remains in the human body longer than nicotine, which increases its biological usefulness (Bocca and Battistini, 2024; Tan et al., 2021). Cotinine has a complex effect on human health. On the one hand, it has been shown that this compound has a beneficial effect on the functioning of the nervous system, while some studies indicate negative effects such as sleep disorders, cognitive impairment, or a link to heart disease (Lei et al., 2023). Most studies on cotinine have been conducted in urine. Higher concentrations of cotinine are found in the urine of classic cigarette smokers compared to nonsmokers and passive smokers (a non-smoker who inhales tobacco smoke from a smoker) (Sharma et al., 2019; Van Overmeire et al., 2016; Jung et al., 2012; Zielińska-Danch et al., 2007; Göney et al., 2016; Jain, 2015; Paci et al., 2018; Yang et al., 2001; Hovanec et al., 2019; Fernandes et al., 2020; Zettergren et al., 2023; Etter and Bullen, 2011). Additionally, the concentrations of this metabolite correlated with the score of the Fagerström Test for Nicotine Dependence scale, higher concentrations being observed in people who had higher scores on this scale (Van Overmeire et al., 2016; Jung et al., 2012). In addition, analysis using the ROC curve showed that urinary cotinine concentrations predicted the high nicotine dependence group with good accuracy (AUC = 0.82; P < 0.001). Interestingly, the cotinine derivative 3-hydroxycotinine was also tested in the urine of smokers, where it showed higher concentrations in smokers. Saliva testing also showed higher concentrations of this metabolite in the unstimulated saliva of smokers relative to non-smokers (Sharma et al., 2019; Etter and Bullen, 2011; Hasan et al., 2024; Mokeem et al., 2018).

In the case of e-cigarette users, higher concentrations of cotinine are also observed in the urine compared to non-users (Pamungkasningsih et al., 2021; Park and Choi, 2019; Zettergren et al., 2023). The highest concentrations of cotinine were found in the urine of people who used e-cigarettes and traditional cigarettes simultaneously, compared to users of e-cigarettes only or traditional cigarettes only (Zettergren et al., 2023). Studies of unstimulated saliva of e-cigarette users, showed higher concentrations of cotinine compared to non-users (Etter and Bullen, 2011; Hasan et al., 2024; Wong et al., 2020; Zhang et al., 2024; Melero-Ollonarte et al., 2023).

Currently, urine studies of HTP users are not available. However, a single study of unstimulated saliva showed higher cotinine concentrations in HTP users compared to non-users (Melero-Ollonarte et al., 2023).

### 4.3 1-Hydroxypyrene

1-Hydroxypyrene is a metabolite of pyrene, and is typically used as a biomarker of polycyclic aromatic hydrocarbon exposure (Ifegwu et al., 2012). Exposure to this compound has been linked to carcinogenic, mutagenic, genotoxic, and teratogenic effects (Yadav et al., 2023). Most studies related to 1-Hydroxypyrene have been conducted on the urine of cigarette smokers. These studies showed higher concentrations of this compound in the urine of smokers compared to non-smokers (Li et al., 2000; Hecht et al., 2004; Zhou et al., 2018; Yadav et al., 2023; Lee and Byeon, 2010; Choosong et al., 2014; Van Rooij et al., 1994; Rodríguez-Rabassa et al., 2018) 4; (Kurniasari et al., 2019; Feng et al., 2024). In addition, the concentration of this metabolite was dependent on the number of cigarettes smoked - the higher the number of cigarettes smoked, the higher the concentration of 1-Hydroxypyrene in urine. In contrast, a decrease in urinary concentrations of this metabolite is observed in individuals who have reduced or quit smoking (Lee and Byeon 2010; Choosong et al., 2014; Hecht et al., 2004) There are currently no studies on saliva.

According to studies by Feng et al. (2024) and Tarassova et al. (2024), smokers of classic cigarettes have higher concentrations of 1-Hydroxypyrene in their urine compared to e-cigarette users. This indicates that e-cigarette users are less likely to be exposed to polycyclic aromatic hydrocarbons present in the smoke.

### 4.4 4-(methylnitrosamino)-1-(3-pyridyl)-1-butanol (NNAL)

4-(Methylnitrosamino)-1-(3-pyridyl)-1-butanol (NNAL) is a specific nitrosamine formed during cigarette smoking (the nitrosation reaction of nicotine and other tobacco alkaloids). NNAL is a potent carcinogen, especially of the lungs and pancreas (Bou Fakhreddine et al., 2014). It is also the most studied nitrosamine among cigarette smokers. Smokers of classic cigarettes have higher urinary NNAL concentrations compared to non-smokers (Carmella et al., 2003; Hou et al., 2012; Benowitz et al., 2018; Xia et al., 2011; Kavvadias et al., 2009). Importantly, according to the work of Kavvadias et al. (2009), NNAL concentrations correlated with factors such as the number of cigarettes smoked per day, salivary cotinine and urinary nicotine concentrations. Interestingly, a single study conducted by Dai et al. (2022) showed that smokers of conventional cigarettes had comparable NNAL concentrations to non-smokers. NNAL concentrations were also studied in the urine of users of other forms of nicotine-e-cigarettes and HTP. According to the work of Xia et al. (2021), HTP users have the highest urinary NNAL concentrations compared to e-cigarette users and regular smokers. This is not consistent with the work of Benowitz et al. (2018) who showed that it is smokers of classic cigarettes have higher urinary NNAL concentrations compared to HTP users. On the other hand, a single study conducted by Dai et al. (2022) found no differences in NNAL concentrations in HTP and e-cigarette users compared to non-smokers. Unambiguously determining the differences in urinary NNAL concentrations in users of different forms of tobacco requires further research, including on saliva. A study by Cartanyà-Hueso et al. (2019) and found that smokers of rolling cigarettes themselves had higher salivary NNAL concentrations compared to smokers of ready-made cigarettes. Importantly, a study conducted by Edmiston et al. (2022) showed that e-cigarette users have higher concentrations of NNAL in their saliva compared to non-users.

### 4.5 Thiocyanate

Cyanides are formed when tobacco and its additives are burned. Chronic exposure to large amounts of cyanide is associated with headaches, dizziness, tremors, and blurred vision (Lachowicz et al., 2024). They are main irritants in tobacco smoke. In the body, cyanides are partially detoxified to thiocyanates (SCN-) by the enzyme rhodanese in the liver (Feng et al., 2024). Higher concentrations of thiocyanate are found in the urine of cigarette smokers compared to non-smoking patients (Rahimi et al., 2018; Buratti et al., 1997; Rodríguez-Rabassa et al., 2018; Mathiaparanam et al., 2023; Jain, 2016; Angelova et al., 2012; Narkowicz et al., 2018; Borgers and Junge, 1979, Scherer, 2006). Importantly, according to a study by Angelova et al. (2012), the amount of thiocyanate in urine increases with the number of cigarettes smoked per day. Saliva studies have shown that cigarette smokers have higher thiocyanate concentrations compared to non-smokers (Cartanyà-Hueso et al., 2019; Angelova et al., 2012; Scherer, 2006; Toraño and Van Kan, 2003; Borgers and Junge, 1979; Glatz et al., 2001; Galanti, 1997; Rodríguez-Rabassa et al., 2018; Aggarwal, et al., 2013; Flieger et al., 2019; D’Cruz and Benny, 2018; Prakruthi et al., 2018; Benny and D’Cruz, 2020). Also, higher salivary thiocyanate concentrations are found in pregnant smokers (Benny and D’Cruz, 2020). Similar to urine studies, saliva studies have shown that thiocyanate concentration increase with the number of cigarettes smoked per day and the duration of smoking (Galanti, 1997; Rodríguez-Rabassa et al., 2018).

A single study of the saliva of e-cigarette users showed that users of this form of tobacco had higher concentrations of thiocyanates in unstimulated saliva compared to non-users of e-cigarettes. However, these concentrations were lower than those of regular cigarette smokers but still higher than those of non-smokers (Aggarwal et al., 2013). Currently, no one has studied cyanide concentrations in the saliva or urine of HTP users, which indicates the need for such studies.

## 5 Cytokines and chemokines as exposure biomarkers in smokers

### 5.1 Interleukin 1β (IL-1β)

It is one of the most important pro-inflammatory cytokines, produced by monocytes and macrophages. In addition, it mediates cellular phenomena, an increase in the production of other pro-inflammatory cytokines and an increase in body temperature (Satoh et al., 2015). Currently, there is only one study on the urine of smokers. A study by Farrell et al. (2023) found that IL-1β concentrations in the urine of smokers and non-smokers were similar and there were no differences between the study groups. Data on saliva of classic cigarette smokers are conflicting. Some studies show that smokers relative to non-smokers have higher concentrations of IL-1β in unstimulated saliva (Farrell et al., 2023; Kamal and Shams, 2022). On the other hand, there are studies that show that smokers and non-smokers have similar concentrations of IL-1β in unstimulated saliva (Zięba et al., 2024; Alhumaidan et al., 2022).

Similar to the urine of classic smokers, the urine of e-cigarettes users showed no significant statistical differences with the group of non-users (Farrell et al., 2023). Higher concentrations of IL-1β are found in the unstimulated saliva of e-cigarette users compared to non-cigarette smokers (Mokeem et al., 2018; Kamal and Shams, 2022; Singh et al., 2019). However, it should be noted that according to some studies, e-cigarette users had lower concentrations of IL-1β in unstimulated saliva relative to non-users (Zięba et al., 2024) or that, concentrations between the two groups were comparable (Verma et al., 2021; Rodríguez-Rabassa et al., 2018).

### 5.2 Interleukin 4 (IL-4)

Interleukin 4 (IL-4) has anti-inflammatory effects; its biological function is related to inhibition of interferon gamma. It is also involved in processes related to allergy formation (Keegan et al., 2021). There is only one single study on the saliva of classic cigarette smokers which showed that smokers have higher concentrations of IL-4 in unstimulated saliva compared to non-smokers (Keegan et al., 2021). Currently, there are no other reports on both saliva and urine of smokers of various forms of tobacco such as e-cigarettes and HTPs.

### 5.3 Interleukin 6 (IL-6)

IL-6 has multifunctional effects based on activation of lymphocytes, inflammation, and increased production of other pro-inflammatory compounds (Liu et al., 2016; Yousif et al., 2021). There is currently one study on urine testing. Classic smokers had higher concentrations of IL-6 in their urine compared to non-smokers (Farrell et al., 2023). According to most available studies, there are no differences in IL-6 levels in the unstimulated saliva of smoking and non-smoking patients (Tylutka et al., 2024; Singh et al., 2019; Verma et al., 2021; Rodríguez-Rabassa et al., 2018), although one study indicates that classic cigarette smokers have higher IL-6 concentrations in unstimulated saliva compared to non-smokers (Mokeem et al., 2018).

E-cigarette users are found to have higher concentrations of IL-6 in urine compared to non-users (Farrell et al., 2023). Similar observations are also found in saliva, where users of e-cigarettes have higher concentrations of IL-6 in unstimulated saliva compared to the unstimulated saliva of non-users (Mokeem et al., 2018).

### 5.4 Interleukin 8 (IL-8, CXCL8)

IL-8 is a potent chemotactic factor for neutrophils. In addition to physiological phenomena, it is also involved in a number of pathological phenomena (Matsushima et al., 2022; Punyani and Sathawane, 2013).

A study of the urine of classic cigarette smokers showed no difference in IL-8 concentrations between the smoking and non-smoking groups (Farrell et al., 2023). Saliva data, on the other hand, are inconsistent. According to studies by Frasheri et al. (2022) and Karaaslan et al. (2020), smokers have lower Il-8 concentrations compared to non-smokers. One study by Zieba et al. (2024) showed that there are no differences in IL-8 concentrations in the unstimulated saliva of smokers and non-smokers. However, on the other hand, according to studies by Sahibzada et al. (2023) and Amirthalingam et al. (2023) smokers have higher IL-8 concentrations in unstimulated saliva compared to non-smokers. Such contradictory reports demonstrate the need to re-examine IL-8 concentrations in the saliva of smokers compared to non-smokers in order to unambiguously determine the clear directions of changes in the concentrations of this chemokine.

In the case of e-cigarettes, studies are also mutually exclusive. Some studies indicate that in the unstimulated saliva of e-cigarette users, IL-8 levels are higher than in the saliva of non-users (Karaaslan et al., 2020), while on the other hand, a study by Zieba et al. (2024) found that e-cigarette users have lower IL-8 concentrations compared to non-users.

One study also looked at HTP, and lower concentrations of IL-8 were found in the unstimulated saliva of HTP users compared to non-users of this form of smoking (Zięba et al., 2024).

### 5.5 Interleukin 10 (IL-10)

Interleukin 10 (IL-10) has anti-inflammatory properties; its action is based on inhibition of the synthesis of pro-inflammatory cytokines (Carlini et al., 2023).

Data on changes in IL-10 concentrations are quite scarce. Currently, we have only two studies - one on urine and the other on saliva. In the urine of traditional cigarette smokers, higher concentrations of IL-10 are found compared to non-smokers (Prakruthi et al., 2018), while in unstimulated saliva, lower concentrations of IL-10 are found in smokers compared to non-smokers (Suzuki et al., 2016).

For users of e-cigarette, urine reports are conflicting. Some studies indicate that users have lower urinary IL-10 concentrations compared to non-users (Singh et al., 2019) however, a single study reports that urinary IL-10 concentrations of e-cigarette users and non-users do not differ between groups (Prakruthi et al., 2018). In the case of unstimulated saliva, all studies found higher IL-10 concentrations in e-cigarette users compared to non-users (Suzuki et al., 2016; Zięba et al., 2024).

One study also found differences in IL-10 concentrations in HTP users - higher concentrations of IL-10 are found in the unstimulated saliva of users of this form of tobacco compared to non-smokers (Zięba et al., 2024).

### 5.6 Interleukin 13 (IL-13)

IL-13 is a cytokine associated with the action of eosinophils and mediates allergic reactions. The biological effects of this cytokine are associated with anti-inflammatory responses (Marone et al., 2019). Cigarette smokers have been found to have higher concentrations of IL-13 in their urine compared to non-smokers (Tanni et al., 2010; Farrell et al., 2023). Unlike urine studies, saliva studies are inconsistent. Rodríguez-Rabassa et al. (2018) found higher levels of IL-13 in the unstimulated saliva of smoking patients compared to non-smoking patients. However, on the other hand, some studies found no difference in IL-13 concentrations in unstimulated saliva of non-smoking and smoking patients (Suzuki et al., 2016). This shows, for the necessity of re-testing IL-13 in the saliva of smokers.

The concentrations of IL-13 in the urine of patients who use e-cigarettes, do not differ from the concentrations of IL-13 in the urine of non-users (Farrell et al., 2023). In contrast, studies of unstimulated saliva showed that e-cigarette users had higher IL-13 concentrations compared to non-users (Rodríguez-Rabassa et al., 2018).

In the case of urine studies of HTP users, we only have a single study that showed that concentrations of this interleukin were equal in HTP users and non-users (Farrell et al., 2023). Saliva studies are conflicting - according to some studies, HTP users have higher concentrations of IL-13 in unstimulated saliva compared to non-users (Farrell et al., 2023; Kamal and Shams, 2022). IL-13 concentrations in the unstimulated saliva of HTP users and non-users were comparable, according to studies by Barbieri et al. (2011), Shireen et al. (2022) and Zięba et al. (2024).

### 5.7 Interleukin 17 (IL-17)

Interleukin 17 (IL-17) is a pro-inflammatory cytokine, increases the production of other inflammatory mediators, and is also involved in processes associated with autoimmune diseases (Roman, 2023; Huangfu et al., 2023). IL-17 in the body fluids of smokers is currently very poorly studied. One study found that IL-17 levels in unstimulated saliva were higher in smokers (Javed et al., 2020), while two other studies showed that there were no differences in IL-17 levels in smokers and non-smokers (Rodríguez-Rabassa et al., 2018; Ponce-Gallegos et al., 2020).

### 5.8 Interleukin 23 (IL-23)

Interleukin 23 (IL-23) is a cytokine that strongly influences the release of acute phase proteins and affects T-cell proliferation and cytotoxicity (Krueger et al., 2024). The number of available studies on IL-23 is limited. Urine studies of smokers showed that smokers had higher concentrations of IL-23 than non-smokers (Barjaktarevic et al., 2016). Saliva studies have also shown that smokers have higher concentrations of this interleukin than non-smokers (Javed et al., 2020; Kroening et al., 2008).

### 5.9 Tumor necrosis factor Alpha (TNF-α)

Tumor Necrosis Factor Alpha (TNF-α) is a pro-inflammatory cytokine, mainly responsible for stimulating apoptosis and necrosis (Grunwald et al., 2024). Currently, there are no scientific data on TNF-α concentrations in the urine of smoking patients. Several studies, refer to saliva where no differences were found in the concentrations of this cytokine between smoking and non-smoking groups (Aggarwal et al., 2013; Flieger et al., 2019).

In contrast, the opposite phenomenon is observed in e-cigarette users - e-cigarette users had higher concentrations of TNF-α compared to non-e-cigarette users (Suzuki et al., 2016; Belkin, et al., 2023; Pushalkar et al., 2020; Sinha et al., 2020).

HTP users also have higher concentrations of TNF-α in unstimulated saliva compared to non-users of this form of tobacco (Zięba et al., 2024).

### 5.10 Monocyte chemoattractant protein 1 (MCP-1)

MCP-1 is a chemokine with potent chemoactivating effects directed specifically at immune cells. Currently, we do not have any studies on MCP-1 concentrations in smoking patients. Data on saliva studies in users of various forms of tobacco are unequivocal. In smokers of classic cigarettes and e-cigarettes and HTP users, lower concentrations of MCP-1 are observed in unstimulated saliva compared to non-smokers and non-users (Matsushima et al., 2022; Sgambato et al., 2018).

### 5.11 Transforming growth factor β (TGF-β)

Transforming Growth Factor Beta (TGF-β) exerts antiproliferative activity, induces apoptosis, and participates in the regulation of tissue repair processes (Aggarwal et al., 2013). Currently, we do not have studies that determine TGF-β concentrations in the urine of tobacco users. Users of classic cigarettes have higher TGF-β concentrations in unstimulated saliva compared to non-users [96]. E-cigarette smokers also have higher concentrations of TGF-β in unstimulated saliva than in the saliva of non-smokers (Kamal and Shams, 2022).

## 6 Oxidation-reduction system

### 6.1 Enzymes of the oxidation-reduction system

Antioxidant enzyme activity was measured only in unstimulated saliva. The first enzyme described is glutathione peroxidase (GPx), an enzyme that reduces hydrogen peroxide and organic peroxides. Studies on GPx in classic cigarette smokers have been inconsistent. In unstimulated saliva, higher (Arbabi-Kalati et al., 2017; Saggu et al., 2012; Jenifer et al., 2015) or lower (Kroening et al., 2008) activity of this enzyme was found. Some studies also indicate that GPx activity in unstimulated saliva was equal in smokers and non-smokers (Zappacosta et al., 2002).

Superoxide dismutase (SOD) is an oxidoreductase enzyme that catalyzes the dismutation of superoxide anion radicals into molecular oxygen and hydrogen peroxide, which is subsequently further degraded by other antioxidant enzymes. All studies show that lower SOD activity is found in the unstimulated saliva of cigarette smokers compared to the unstimulated saliva of non-smoking patients (Saggu et al., 2012; Syed et al., 2021; Baharvand et al., 2010; Yadav et al., 2020). HTP users are found to have higher SOD activity (Suvarna et al., 2023) or lower SOD activity (Kroening et al., 2008) in unstimulated saliva compared to non-HTP users.

Catalase is an enzyme that breaks down hydrogen peroxide into oxygen and into water. Studies on catalase are limited to classic cigarette smokers only. All studies have shown that smokers have lower catalase activity in unstimulated saliva compared to non-smoking patients (Singh et al., 2019; Balasubramaniam and Arumugham, 2022; Ahmadi-Motamayel et al., 2018)

### 6.2 Other components of the oxidation-reduction system

#### 6.2.1 Malondialdehyde

Malondialdehyde (MDA) is a byproduct of lipid peroxidation, generated through the reaction of reactive oxygen species (ROS) with polyunsaturated fatty acids. It is widely recognized as a biomarker of oxidative stress. High MDA concentrations are associated with a number of diseases such as cardiovascular and nervous system diseases, as well as cancer and inflammatory diseases (Toto et al., 2022). All studies included unstimulated saliva of patients who smoke traditional cigarettes - smokers had higher concentrations of MDA in unstimulated saliva compared to non-smokers (Zięba et al., 2024; Demirtaş et al., 2014; Mohammed et al., 2014). One study looked at e-cigarette users. In the unstimulated saliva of such individuals, higher concentrations of MDA are found compared to non-users, and equal results are also found in HTP users - non-users had higher concentrations of MDA than users (Zięba et al., 2024).

#### 6.2.2 4-Hydroxynonenal (4-HNE)

4-Hydroxynonenal (4-HNE) is a product of lipid peroxidation, formed by the oxidation of polyunsaturated fatty acids. The effect of this compound has been linked to carcinogenesis, Alzheimer’s disease, and inflammatory diseases (Shoeb et al., 2014). In smokers of classic cigarettes, higher concentrations of 4-HNE are found, compared to the urine of non-smoking patients (Bozkuş et al., 2017; Shoeb et al., 2014; Li et al., 2022; Eskelinen et al., 2022; Zięba et al., 2024; Žarković et al., 2024). Studies on saliva are unavailable.

In e-cigarette users, tests have found higher levels of urinary 4-HNE than in those who do not use this form of tobacco (Shoeb et al., 2014; Li et al., 2022; Eskelinen et al., 2022; Zięba et al., 2024; Žarković et al., 2024). Higher 4-HNE is found in unstimulated saliva compared to non-users (Majid, 2024).

#### 6.2.3 Uric acid

Although uric acid contributes significantly-up to 50%-to the blood’s antioxidant capacity, hyperuricemia is simultaneously recognized as an indicator of oxidative stress. High concentrations of uric acid are associated with gout, hypertension, and insulin resistance (Roman, 2023). Urine analysis indicates that cigarette smokers have higher uric acid concentrations compared to non-smokers (Miguel et al., 2022). Also, a urine analysis of pregnant smoking patients showed that females smokers have higher concentrations of uric acid in their urine (Lain et al., 2005). However, according to a study by Hanna et al. (2008), no differences were found between uric acid concentrations in smokers and non-smokers. Studies of unstimulated saliva are also contradictory. According to some of the studies, uric acid concentrations in unstimulated saliva are higher in smokers (Zarabadipour et al., 2022), however, according to a study by Miguel et al. (2022), uric acid concentrations were equal in the unstimulated saliva of smokers and non-smokers. A summary of data on changes in biomarkers in the saliva and urine of smokers, is presented in Table 1.

## 7 Future perspectives

The present study demonstrates that knowledge of exposure biomarkers determined in saliva and urine in smokers is sparse. Future studies should focus on the determination of exposure biomarkers in saliva and urine in more patients. Studies should also include the group of patients who use nicotine replacement therapy. Currently, medicine does not have this type of research. It will also be important to correlate exposure biomarker concentrations with nicotine dependence questionnaires such as the Fagerström scales (Mi et al., 2025). Depression is associated with smoking cigarettes and other tobacco products. The anxiolytic and antidepressant effects of smoking are frequently reported by cigarette smokers. Smoking is a behavior that increases the risk of depression (Wu et al., 2023; Fluharty et al., 2017). In addition, depressed patients tend to turn to various forms of nicotine use. Therefore, it also seems expedient to correlate biomarkers of exposure from urine and saliva with the frequency and type of tobacco used with depression scales such as the Hamilton Depression Scale (Munafò and Araya, 2010). Studies of this type may demonstrate future applicability to smokers of various forms of tobacco.

## 8 Limitations

First, the amount of scientific data included in the paper is relatively small. This is due to the fact that this topic is currently poorly understood. However, this work aggregates all available data on studies of biomarker concentrations in the urine and saliva of smokers of various forms of tobacco.

Second, our work does not cover issues related to nicotine replacement therapy and other forms of tobacco use such as snus - pouches with nicotine placed under the gum. This is because such studies have not currently been conducted.

Third, in our study we limited ourselves to urine and saliva only, and did not consider other body fluids and tissues such as blood. This was intentional, as we wanted to focus on only non-invasively collected types of study material. Blood, despite its relative ease of acquisition in collection, continues to be a minimally invasive procedure, so we did not consider work on serum or plasma when preparing this work.

## 9 Discussion

Data on biomarkers of exposure in smokers of various forms of tobacco are currently sparse and often mutually exclusive. Assessment of biomarkers of exposure has many medical benefits. Especially biomarkers collected by non-invasive methods, i.e., saliva and urine. According to the data we collected, concentrations of exposure biomarkers in saliva and urine of users of various forms of tobacco, can be elevated, reduced or remain unchanged from non-smokers. It is unfortunate that most of the data are for smokers of regular cigarettes; the number of reports on other forms of smoking is sparse. Understanding the exact relationship between smoking and changes in biochemical indicators will make it possible to determine the exact biological changes that occur in the human body under the influence of smoking various forms of tobacco, and to link them to different types of diseases, including mental illnesses such as depression.

Although biomarkers of exposure can also be determined in other types of biological material such as tears, sweat, hair or fingernails, these are already methods that require medical procedures such as the administration of pharmacological agents or the use of treatments such as iontophoresis (tears or sweat) or may involve minimal tissue disruption (fingernails, hair). Currently, there are no studies of biomarkers of exposure in this type of material in users of various forms of tobacco, which provides a broad opportunity for such studies to be performed in the future. This will allow a better understanding of the relationship between oxidative stress, inflammatory cytokine effects and smoking.

Biomarkers whose concentrations increase in users of various forms of tobacco compared to non-smokers or no-users include nicotine, cotinine, NNAL, IL-6, IL-13, IL-23, 4-HNE and uric acid. In the case of cotinine, nicotine and NNAL, these are classic biomarkers of cigarette smoking which indicates their effectiveness in assessing exposure to this form of stimulant. IL-6 and IL-23 are pro-inflammatory cytokines (Yadav et al., 2023). An increase in their concentration in the urine of users of various forms of tobacco, may indicate a developing inflammation in the body of smokers of various forms of tobacco. In contrast to IL-6 and IL-23, IL-13 is a cytokine with anti-inflammatory properties (Yadav et al., 2023). This cytokine is mainly involved in reactions of an allergenic nature, which may suggest that users of various forms of tobacco may introduce allergen-like molecules into the body, in addition to typical components of smoke (Yousif et al., 2021). In the case of high concentrations of 4-HNE and uric acid, it can be suggested that using various forms of tobacco is associated with the induction of oxidative stress at such a level that biomarkers of this phenomenon make their way up to the urine.

In the case of saliva testing, most of the biomarkers we described have higher concentrations in smokers compared to non-smokers or users compared to non-users. This has to do with the fact that different smoking regimes directly come into contact with the oral cavity which results in biochemical changes in saliva. In addition to classic biomarkers such as nicotine or cotinine, higher concentrations are found in saliva - of compounds such as IL-6 or TGF-beta. Studies of saliva have shown interesting relationships. As mentioned earlier, IL-6 is a pro-inflammatory cytokine, its concentrations in regular cigarette smokers are comparable to non-smokers, however, higher concentrations of this compound are found in e-cigarette users compared to non-users. This may indicate higher inflammation, in e-cigarette users, compared to users of regular cigarettes. This may be related, for example, to the induction of inflammation by microplastic whose formation is induced by heating the device (Yang et al., 2001). In the case of IL-13 and TNF-α, it is also found that e-cigarette and HTP users have higher concentrations of these cytokines compared to users of conventional cigarettes. Higher concentrations of IL-13 may again indicate the introduction of allergen-like compounds, while TNF-α indicates stronger inflammation in HTP and e-cigarette users. An interesting phenomenon is also observed for IL-10. Smokers of regular cigarettes are found to have lower concentrations of Il-10 compared to non-smokers. In users of e-cigarettes and HTPs, the opposite observation is found - users had lower concentrations of IL-10 in their saliva. It is not known what the reason for this observation is, however, it is postulated that the electronic system containing various types of chemical material (such as plastic) present in HTPs and e-cigarettes stimulates the production of IL-10. Nevertheless, using both e-cigarettes and HTPs has an effect on disrupting the homeostasis of cytokines in the oral cavity of smokers just as much as classic cigarettes.

Although smoking is associated with the risk of developing numerous diseases, mental illnesses such as Major Depressive Disorder (MDD) are rarely given adequate attention. Tobacco use is linked to the inhibition of Monoamine Oxidase (MAO) activity. This promotes the effects of nicotine and contributes to the addictive properties of cigarettes (Zappacosta et al., 2002). The mechanism by which smoking inhibits MAO is not fully understood; however, both MAO-A and MAO-B are particularly affected. Inhibition of MAO activity is associated with the alleviation of depressive symptoms, in line with the monoamine theory. MAO is responsible for the breakdown of neurotransmitters in the brain: norepinephrine, serotonin, dopamine, and tyramine. MAO inhibitors (MAOIs) increase the activity of these neurotransmitters in the brain, although this effect is temporary (Zarabadipour et al., 2022).

On the other hand, smoking is associated with increased activity of various inflammatory mediators and oxidative stress. It has been shown that one of the pathomechanisms of depression involves an imbalance in the production of pro-inflammatory and anti-inflammatory cytokines, with an increase in pro-inflammatory cytokines and a simultaneous decrease in anti-inflammatory ones. The brain has weak antioxidant defenses and a high rate of oxygen consumption, making it particularly susceptible to oxidative stress. Increased ROS (reactive oxygen species) production is associated with higher levels of pro-inflammatory cytokines such as IL-1β, TNF-α, and IL-6 (Yang et al., 2001; Zarabadipour et al., 2022). These pro-inflammatory cytokines have the potential to suppress frontal lobe activity, as evidenced by functional magnetic resonance imaging (fMRI) findings, thereby contributing to the manifestation of depressive symptoms (Žarković et al., 2024)

According to the data collected by our team, increases in pro-inflammatory cytokines and decreases in anti-inflammatory cytokines are not always detectable in urine or saliva. It is very likely that such changes are only observable in serum, plasma, or cerebrospinal fluid, suggesting that the alterations are too subtle to be detected in saliva and urine. Furthermore, according to the depression theory, its onset must involve several interrelated pathomechanisms, such as genetic or neuroplasticity-related factors (Zarabadipour et al., 2022). The Fagerström scales may be useful in assessing the impact of smoking on depression and its association with oxidative stress and pro-inflammatory cytokines.

In conclusion, the effects of using various forms of tobacco on biochemical changes in the human body are currently poorly studied. In particular, studies on e-cigarettes and HTP are lacking. The most convenient form of research material is collected by non-invasive methods; these include urine and saliva. Most of the studies on saliva and urine of users of various forms of tobacco firstly concern classic cigarettes and secondly are often contradictory. This indicates the need to make determinations of this type in users of various forms of tobacco. In addition, given that smoking affects the balance of pro-inflammatory/anti-inflammatory cytokines and has a role in the induction of oxidative stress, it seems expedient to link smoking of various forms of tobacco to both depression, which may have a biochemical and oxidative stress-related basis.

## 10 Conclusion

Although the number of traditional cigarette smokers has fallen, the number of users of other forms of tobacco, such as e-cigarettes or HTPs, is steadily increasing. Toxicity and the degree of nicotine dependence are influenced by the way nicotine is delivered - the forms of smoking. Exposure biomarkers are used to assess the effects and degree of exposure to tobacco products. Their concentrations in the smoker’s body are modulated by smoking different forms of tobacco. Although medicine already has classical biomarkers of tobacco smoke exposure such as cotinine, 1-Hydroxypyrene or NNAL they are characterized by limited specificity. Therefore, it is advisable to look for other biomarkers of exposure, especially those whose analysis can be performed in material collected non-invasively such as saliva or urine. According to the data we have collected, users’ various forms of tobacco affect the concentrations of both classical biomarkers and potential biomarkers. However, data are often sparse and often contradictory. Therefore, it is important to perform future thorough studies on the concentrations of exposure biomarkers in users of different forms of nicotine exposure.

## Abbreviations

4-HNE, 4-Hydroxynonenal; AUC, Area Under the Curve; CXCL8, C-X-C Motif Chemokine Ligand 8; GPx, glutathione peroxidase; HTPs, heated tobacco products; IL-10, Interleukin 10; IL-13, Interleukin 13; IL-17, Interleukin 17; IL-1β, Interleukin 1β; IL-23, Interleukin 23; IL-4, Interleukin 4; Il-6, Interleukin 6; Il-8, IL-8 interleukin 8; MCP-1, Monocyte Chemoattractant Protein 1; MDA, malondialdehyde; NNAL, 4-(methylnitrosamino)-1-(3-pyridyl)-1-butanol; SOD, superoxide dismutase; TGF-β, Transforming Growth Factor β; TNF-α, Tumor Necrosis Factor Alpha.
